# 25(OH)D_3_ Affects the Maturation and Function of Mouse Bone Marrow-Derived Dendritic Cells Stimulated by *Mycobacterium* Bovis BCG

**DOI:** 10.1371/journal.pone.0048062

**Published:** 2012-11-05

**Authors:** Hui-feng Yang, Ze-hua Zhang, Liang-bi Xiang, Kang-lai Tang, Fei Luo, Chun-yu Liu, Jian-bo Zhou, Jin-qing Li, Jian-zhong Xu

**Affiliations:** 1 Department of Orthopedic Surgery, Southwest Hospital, Third Military Medical University, Chongqing, People’s Republic of China; 2 Department of Orthopedic Surgery, General Hospital of Shenyang Military Command, Shenyang, People’s Republic of China; Institute of Infectious Diseases and Molecular Medicine, South Africa

## Abstract

It has been shown that vitamin D deficiency increases an individual’s susceptibility to tuberculosis (TB). However, very little is known about the effect of vitamin D on the immune response to *Mycobacterium tuberculosis* (*M. tb*) in dendritic cells (DCs). Because DCs play an important role in TB infection, we investigated the phenotypic characteristics and functional capabilities of mouse bone marrow-derived dendritic cells (BMDCs) after stimulation with Bacillus Calmette-Guérin (BCG) in the presence or absence of 25(OH)D_3_(100 nM). Bone marrow cells from mice were cultured with GM-CSF (20 ng/ml) and were then treated with 25(OH)D_3_ for 7 days. On day 6, 5 µg/ml of BCG (≥1.0×10^6^ CFU/mg) was added to the cells for 24 hours, and on day 7, the non-adherent cells were harvested for phenotypic and functional analyses. After incubation with 25(OH)D_3_, the expression levels of MHC-II and CD86 on the surface of the dendritic cells (DCs) and the ability of the DCs to stimulate proliferation of allogeneic mixed lymphocytes were lower than control cells (*p*<0.05). Furthermore, the level of Interleukin (IL) -4 secreted by the BMDCs in the 25(OH)D_3_ culture was lower than that in the control culture (*p*<0.01). However, the BMDCs cultured with 25(OH)D_3_ produced significantly higher levels of IL-2, IL-6, IL-10 and interferon gamma(IFN-γ) than those in the control culture (*p*<0.05). These findings suggest that 25(OH)D_3_ modulates the immune response during *mycobacterial* infection by affecting the maturation and function of DCs.

## Introduction

Tuberculosis (TB) is a major global disease that is estimated to kill 1.45 million people in 2010 [1]. Calciferol (Vitamin D) was used to treat tuberculosis in the late 1940s and treatment was rationalised based on its role in the calcification of tuberculosis lesions. With the advent of effective anti-tuberculosis drugs in the mid-1950s, the enthusiasm for treating tuberculosis with vitamin D subsided [2]. However, increasingly more patients with tuberculosis are now infected with drug-resistant strains [3], [4], the infection is harder to treat as normal treatment regimes are ineffective. Thus why the use of vitamin D may be useful in the context of drug resistance. Currently, the effects of vitamin D on TB patients remain controversial. Morcos et al [5] reported that the addition of vitamin D to the standard antitubercular therapy results in higher rates of clinical and radiological improvement. Nursyam et al [6] also reported that there were more TB patients with radiological improvement in the vitamin D group. Recently, Martineau et al [7] found that administration of four doses of 2.5 mg vitamin D_3_ significantly hasten sputum culture conversion in participants with the *tt* genotype of the *TaqI* vitamin D receptor polymorphism. On the contrary, Wejse et al [8] stated that vitamin D does not improve clinical outcome among patients with TB and the trial showed no overall effect on mortality in patients with TB. Furthermore, Sato et al [9] thought that low serum vitamin D level is a good predictor of prolonged clinical course in patients with active pulmonary TB. Besides, Koo et al [10] considered that vitamin D levels do not appear to be associated with the development of TB in the Korean population. The median 25(OH)D concentration decreased after treatment for TB. Therefore, more detailed studies are necessary to further elucidate the role of vitamin D in TB infection.

Vitamin D has been shown to play an important role in the innate and adaptive immune responses to tuberculosis [11]. Recently, many mechanisms by which vitamin D influences the innate immune response to TB have been reported [12], [13], [14]. However, the mechanism by which vitamin D regulates the adaptive immune response remains less well characterized. Dendritic cells (DCs) are the primary cell type that initiates the adaptive immune response against Mycobacterium tuberculosis (*M. tb)* infection [15], [16], [17]. After bacterial uptake, DCs change their behavior, as observed both *in vitro* and *in vivo*, by reducing their phagocytic and/or endocytic capability and initiating expression of immune stimulatory molecules. This process is termed DC maturation, and it is initiated in nonlymphoid tissues where DCs take up antigen (Ag) and continues within secondary lymphoid tissues, particularly the draining lymph nodes (DLNs). Differentiation of immature DCs in the DLNs leads to phenotypic changes into the mature phenotype resulting in expression of high levels of long-lived MHC I, MHC II molecules and other costimulatory molecules (CD80,CD86) [15]. Furthermore, recent data have shown a critical role for DCs in the priming of the CD4+ T cell response to early secretory antigenic target 6-kDa (ESAT-6), thereby suggesting that DCs are essential for the initiation of the adaptive T cell response to human *M. tb* infection [16]. The effect of vitamin D on DC maturation has been extensively studied [18], [19], [20], but very little is known how vitamin D affects DC during *mycobacterial* infection.

**Figure 1:**
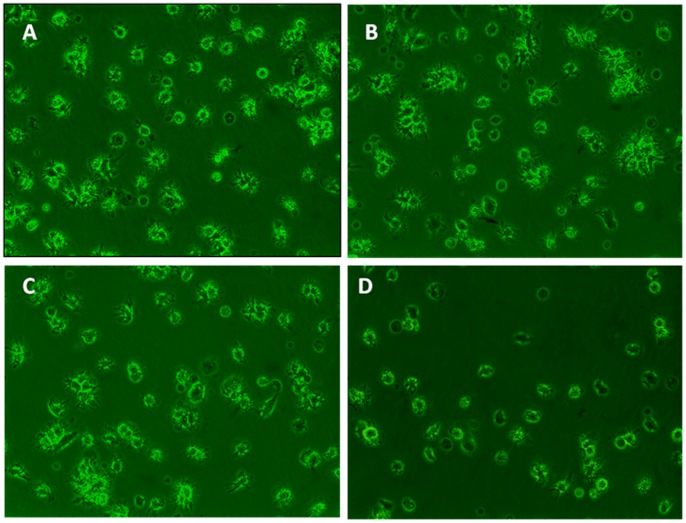
Morphology of BMDCs. The BM cultures were pulsed on day 6 with 5 µg/ml of BCG for 24 h. The phase contrast photographs (200× magnification) were taken directly from the day 7 cultures. Numerous non-adherent mature DCs with large veils are visible in the control, 25(OH)D_3_(100 nM), 1,25(OH)_2_D_3_(10 pM) cultures (Fig. 1A,B,C), but few are in the 1,25(OH)_2_D_3_ (100 nM) cultures (Fig. 1D).

**Figure 2:**
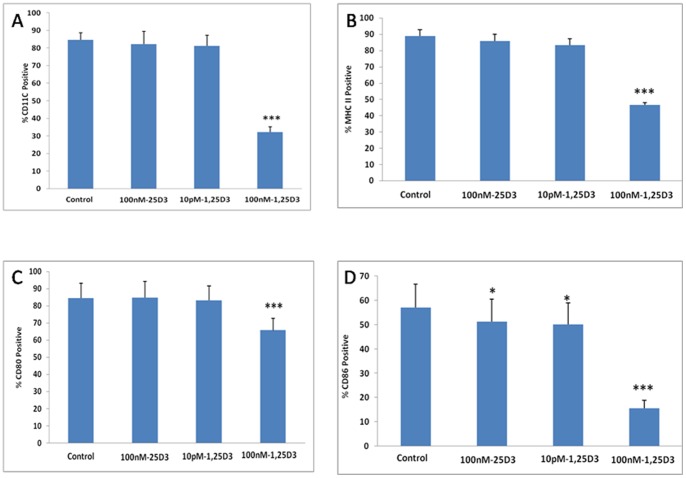
The percentages of CD11C, MHC II, CD80, CD86. A. CD11C; B. MHCII; C. CD80; D. CD86; All the data from five mice (means ± SEM) and were analyzed using ANOVA. *: *p*<0.05, ***: *p*<0.001when compared to control.

Therefore, in this study, we assessed the phenotypic and functional differences of bone marrow-derived dendritic cells (BMDCs) from C57BL/6 mice stimulated with M. bovis BCG in the absence or the presence of 100 nM 25(OH)D_3_.

**Figure 3:**
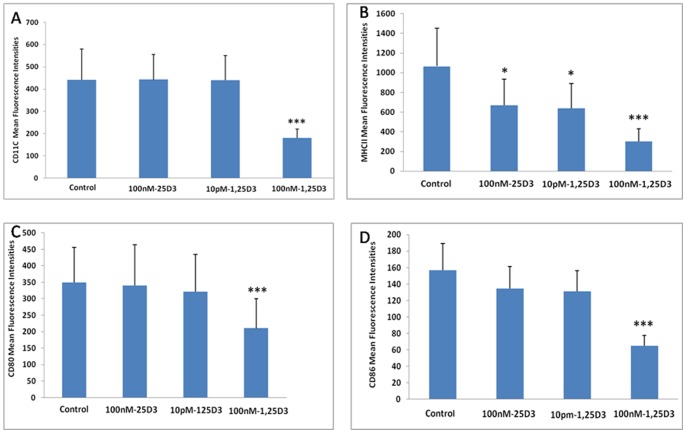
The mean fluorescence intensities for CD11C, MHC II, CD80 and CD86. A. CD11C; B. MHCII; C. CD80; D. CD86. All the data from five mice (means ± SEM) and were analyzed using ANOVA. *: *p*<0.05, ***: *p*<0.001 when compared to control.

**Figure 4:**
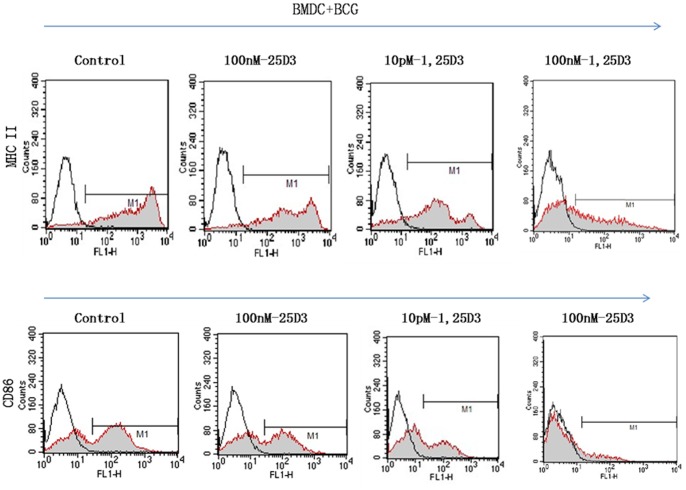
The percentages of MHC II and CD86. Representative flow cytometry histograms of phenotypic markers on −/+25(OH)D_3_, 1,25(OH)2D_3_(10 pM) and 1,25(OH)2D_3_(100 nM)-treated cells stimulated with BCG are illustrated. The open histogram represents isotype-control cells, and the filled histogram represents stained cells.

## Materials and Methods

### Bone Marrow Preparation

All mice were kept under Specific Pathogen-Free (SPF) conditions in our own facilities (Chongqing, People’s Republic of China). All animals were provided by the animal center of the Third Military Medical University and all experiments were approved by the Ethics Committees (The Ethics Committee, Southwest Hospital, Third Military Medical University). The mice were euthanized, and then both the femurs and tibiae of male C57BL/6 mice (6–8 weeks old) were removed and separated from the surrounding muscle tissue using fine scissors and tweezers. Thereafter, the intact bones were immersed in 70% ethanol for 1 min for disinfection and washed with RPMI-1640 medium. Next, both ends of the bones were cut with scissors, and the marrow was flushed out with RPMI-1640 medium using a syringe with a 26-gauge needle. The clusters within the marrow suspension were disrupted by vigorous pipetting. After one wash in RPMI-1640 medium, approximately 2×10^7^ cells were obtained per mouse. Five mice were used for each experiment.

**Figure 5:**
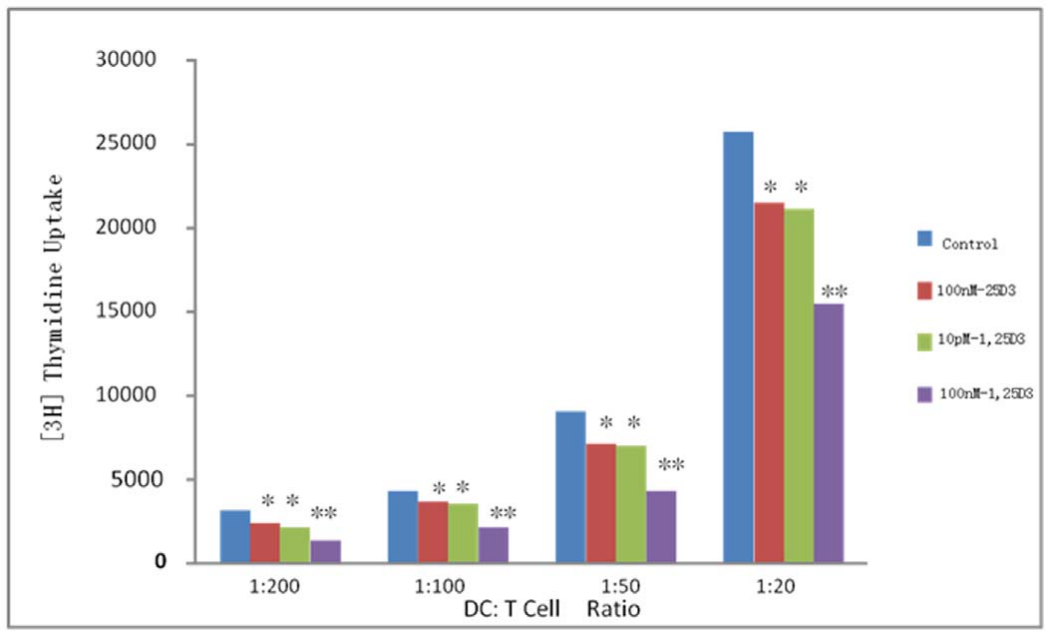
Allogeneic mixed lymphocyte reactions. The allostimulatory capacities of the −/+25(OH)D_3_, 1,25(OH)2D_3_(10 pM) and 1,25(OH)2D_3_(100 nM)-treated BMDC cultures were measured by T cell proliferation rates. T cell proliferation was measured by incorporation of ^3^H-thymidine. The ratios of DCs to T cells are depicted on the x-axis. All the data from five mice (means ± SEM) and were analyzed using ANOVA. *: *p*<0.05, **: *p*<0.01 when compared to control.

### Bone Marrow-derived Dendritic Cells

The method for generating BMDCs with GM-CSF (granulocyte–macrophage colony-stimulating factor) was adapted from a previous publication [21]. The method was modified as follows. The cell culture medium (R10) contained RPMI-1640 (Invitrogen) supplemented with penicillin (100 U/ml, Sigma-Aldrich), streptomycin (100 µg/ml, Sigma-Aldrich), L-glutamine (2 mM, Sigma-Aldrich), 2-mercaptoethanol (50 µM, Sigma-Aldrich), 10 mM HEPES (Invitrogen), and 10% heat-inactivated FBS (PAA, Germany). The cells were plated in 24-well culture plates (5×10^5^/ml). On day 0, the bone marrow cells from each mouse were divided into four populations. The first population was cultured as the control culture. The second population was cultured as 25(OH)D_3_ (100 nM, Sigma-Aldrich)culture. The third population was cultured as 1,25(OH)_2_D_3_ (10 pM, Sigma-Aldrich) culture. The fourth population was cultured as 1,25(OH)_2_D_3_ (100 nM, Sigma-Aldrich) culture. The bone marrow cells were seeded at 2.5×10^5^ cells/well in 0.5 ml of R10 medium containing 20 ng/ml recombinant murine GM-CSF (rm GM-CSF) (≥2×10^7^ U/mg; Peprotech). On day 2, 0.4 ml of the old medium was gently removed, and an equal volume of fresh R10 medium containing 25 ng/ml rmGM-CSF and −/+25(OH)D_3_ (100 nM), 1,25(OH)_2_D_3_ (10 pM), 1,25(OH)_2_D_3_ (100 nM) were added to the four populations respectively. On day 4, 0.25 ml of the old medium was gently removed, and an equal volume of fresh R10 medium containing 20 ng/ml rmGM-CSF and −/+25(OH)D_3_ (100 nM), 1,25(OH)_2_D_3_ (10 pM), 1,25(OH)_2_D_3_(100 nM) were added to the four populations respectively. On day 6, 0.25 ml of the old medium was gently removed and centrifuged at 280×*g* for 10 min at room temperature, and the cells were resuspended in 0.25 ml of fresh R10 containing 20 ng/ml rmGM-CSF and −/+25(OH)D_3_ (100 nM), 1,25(OH)_2_D_3_ (10 pM), 1,25(OH)_2_D_3_ (100 nM) were added to the four populations respectively. This solution was then added back into the original well. To induce complete maturation of the DCs on day 6, 5 µg/ml of BCG (≥1.0×10^6 ^CFU/mg) was added to each well for 24 hours.

**Figure 6:**
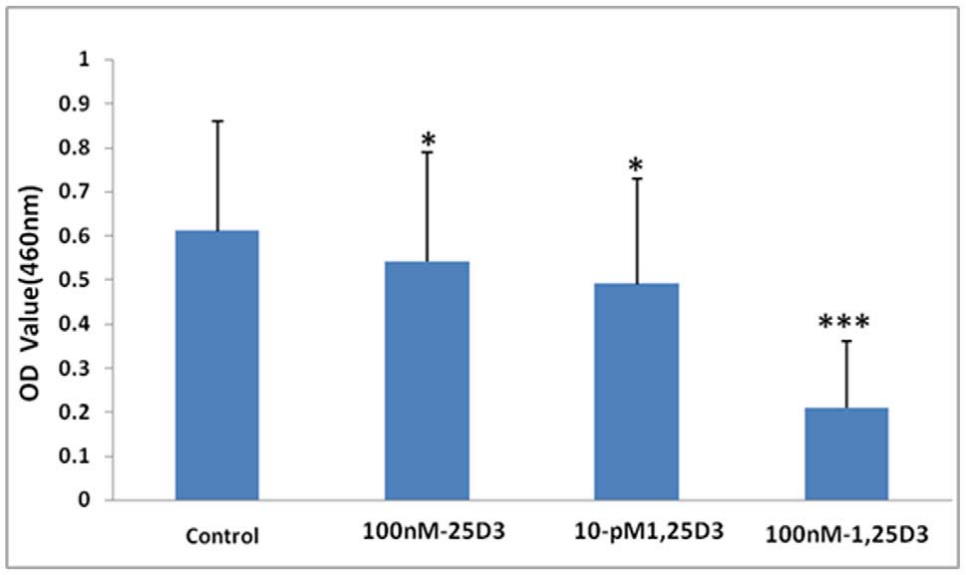
The cell proliferation of BMDCs. The cell proliferation was measured by OD values according to the WST-1 assay and shown for −/+25(OH)D_3_, 1,25(OH)2D_3_(10 pM) and 1,25(OH)2D_3_(100 nM)-treated cultures after BCG stimulation. cell proliferation. All the data from five mice (means ± SEM) and were analyzed using ANOVA. *: *p*<0.05, ***: *p*<0.001 when compared to control.

### Phenotypic Analysis of BMDCs Using Flow Cytometry

After 7 days of maturation +/− vitamin D metabolites, the non-adherent cells were harvested and counted, and 5×10^5^ cells were placed in each tube for staining. The cells were washed twice with PBS. Antibodies specific for CD11C, MHC II, CD80, and CD86 or their isotype controls were added to the tubes, and the tubes were incubated at 4°C for 30 min. The cells were then washed three times with PBS and resuspended in 200 µl of PBS for flow cytometry analysis (FACSCalibur, BD). All of the antibodies used were from the EB Company.

**Figure 7:**
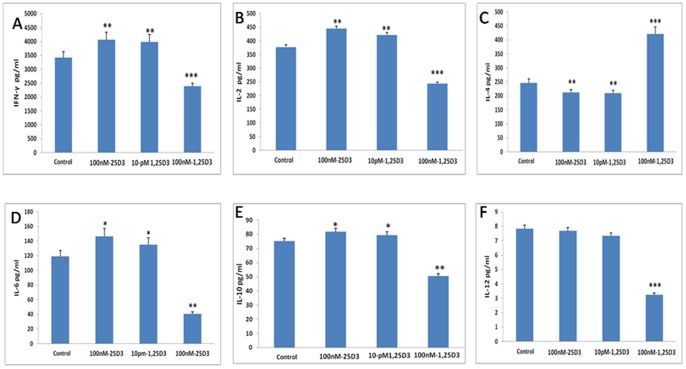
The levels of IFN-γ, IL-2, IL-4, IL-6, IL-10 and IL-12. A. IFN-γ; B. IL-2; C. IL-4; D. IL-6; E. IL-10; F. IL-12. All the data from five mice (means ± SEM) and were analyzed using ANOVA. *: *p*<0.05, **: *p*<0.01,***: *p*<0.001 when compared to control.

### Allogeneic Mixed Lymphocyte Reaction

CD4+ T cells were purified from C57BL/6 spleen cells by magnetic-activated cell sorting (MACS) according to the manufacturer’s instructions (Miltenyi Biotec, Germany). The purity of the CD4+ T cells was between 85 and 95%. In triplicate, 1×10^5^ enriched CD4+ T cells/well were seeded into 96-well flat bottom plates (Falcon) with a titrated number of misogynic C-treated (50 µg/ml, 20 min, 37°C, Sigma-Aldrich) BMDCs. The cells were pulsed with 1 µCi/well of [^3^H]methyl-thymidine (Amersham) overnight for 16 h. The contents of the plates were harvested onto glass fiber filter-mats using an IH-110 harvester (Inotech, Dottikon, Switzerland), and the filters were counted in a 1450 Microplate Counter (Wallac, Turku, Finland).

**Figure 8:**
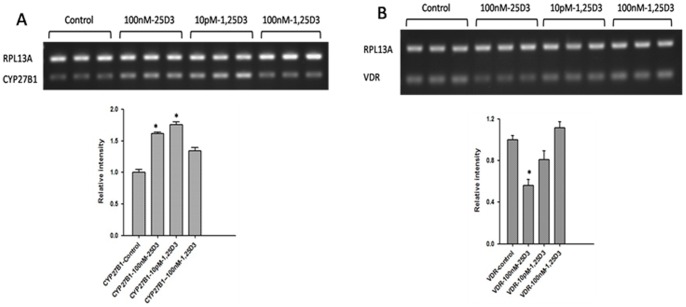
The expression of CYP27B1and VDR. A. The expression of CYP27B1 in 25(OH)D_3_ -treated cells was higher than control-treated cells; But not significantly different between 1,25(OH)_2_D_3_(100 nM)-treated cells and control-treated cells. *: *p*<0.05 when compared to control. B. The expression of VDR in 25(OH)D_3_ and 1,25(OH)_2_D_3_(10 pM)-treated cells was lower than control-treated cells; But The expression of CYP27B1 in 25(OH)D_3_ -treated cells was higher than control-treated cells; But not significantly different between 1,25(OH)_2_D_3_(100 nM)-treated cells and control-treated cells. *: *p*<0.05 when compared to control. The RPL13A in Figure A and B was the housekeeping gene, the relative intensity was the ratio of the expression of target and housekeeping gene.

### BMDC Proliferation

On day 7, the BMDCs stimulated with BCG were collected and counted, and 0.1−5×10^4^ cells/well were added to 96-well plates. The cell proliferation reagent WST-1 (10 µl, Roche) was added to each well, and the plates were incubated for 0.5–4 h. The Optical density (OD) value of the samples were measured at 420–480 nm using a microplate reader, and media were used as the blanks.

### Cytokine Assay

On day 7, the culture supernatants of the four BMDC cultures were collected and stored at −80°C. The levels of interferon gamma (IFN-γ), Interleukin (IL)-2, IL-4, IL-6, IL-10 and IL-12 were determined using enzyme-linked immunosorbent assay (ELISA) kits (R&D Systems, USA) according to the manufacturer’s instructions. The minimum detectable dose of the ELISA kits is typically less than 2.5 pg/mL.

### Reverse Transcriptase Polymerase Chain Reaction (RT-PCR)

To evaluate the expression of the CYP27B1 and VDR, RT-PCR was used. Total RNA was extracted from BMDCs using the RNeasy kit (Qiagen GmbH, Germany) according to the manufacturer’s instructions. Isolated RNA was treated with RNase-free DNase (QIAGEN GmbH) before being reverse transcribed into cDNA. Total RNA was measured by a spectrophotometer (Beckman, Fullerton, CA) at 260 nm and 280 nm. The RNA (1 µg) was reverse transcribed with an oligo (dT) primer using the ThermoScriptTM RT-PCR system (Invitrogen, USA) for cDNA synthesis. For RT-PCR, 1 µl of cDNA template was used for each reaction and sequences were amplified using Taq DNA polymerase. PCR primer for CYP27B1 and VDR were as follows: CYP27B1: forward primer, 5′GCGCCAGCGGGGACGTG 3; reverse primer, 5′GGGCAAAGGCAAACATCTGATCC 3′. VDR: forward primer, 5′CCCCGATTCCAAACCTACCTGCC 3; reverse primer, 5′GGCAGGAGGAGACGGGACACA 3′. RPL13A: forward primer, 5′GCCGAAAGGTGGTGGTTGTAC 3′; reverse primer, 5′ACGCCCCAGGTAAGCAAAC 3′. RPL13A was used to normalize the reactions. Each sample was assessed three times for each gene.

### Statistical Analyses

The data are represented as the means ± SEM. The means of the four groups were tested for statistically significant differences using ANOVA. A *p*-value of less than 0.05 was considered to be statistically significant. Data analyses were performed using SPSS version 13.0.

## Results

### Vitamin D Metabolites Inhibit DC Maturation

The BM cultures were pulsed on day 6 with 5 µg/ml of BCG for 24 h. The phase contrast photographs (200× magnification) were taken directly from the day 7 cultures. Numerous non-adherent DCs with large veils are visible in the control, 25(OH)D_3_(100 nM), 1,25(OH)_2_D_3_(10 pM) cultures (Fig. 1A,B,C), but few are in the 1,25(OH)2D3 (100 nM) cultures (Fig. 1D). The percentages of CD11C-positive cells in the control, 25(OH)D3(100 nM), 1,25(OH)2D3(10 pM), 1,25(OH)2D3 (100 nM) cultures were 84.64%±4.00, 82.28%±7.27(p = 0.85), 81.17%±6.15 (p = 0.79) and 32.25%±3.02 (P<0.001) respectively (Figure 2A). The percentages of cells in the control, 25(OH)D3(100 nM), 1,25(OH)2D3(10 pM), 1,25(OH)2D3 (100 nM) cultures that were positive for the expression of various costimulatory molecules were as follows: MHCII: 88.75%±4. 14, 85.92%±4.05 (p = 0.54), 83.37%±3.96 (p = 0.46) and 46.65%±1.24 (P<0.001) respectively (Figure2B); CD80: 84.51%±8.63, 84.75%±9.37 (p = 0.68), 83.15%±8.48 (p = 0.61) and 65.67±7.12 (P<0.001) respectively (Figure 2C); and CD86: 57.08%±9.55, 51.32%±9.21 (P<0.05), 50.15%±8.76 (P<0.05) and 15.69%±3.12 (P<0.001) respectively (Figure 2D).

The mean fluorescence intensities of the various proteins in the control, 25(OH)D_3_(100 nM), and between 1,25(OH)_2_D_3_(10 pM), 1,25(OH)_2_D_3_ (100 nM) cultures were as follows: CD11C: 441.23±137.95,442.33±113.17(*p* = 0.42), 440.26±110.18 (*p* = 0.40) and 180.65±40.46 (*P*<0.001) respectively (Figure 3A); MHC II: 1065.67±388.89, 670.83±261.81 (*P*<0.05), 640.37±249.62 (*P*<0.05) and 300.12±130.64 (*P*<0.001)respectively (Figure 3B and 4); CD80: 349.43±106.38, 339.73±124.73 (*p* = 0.52), 321.65±112.79 (*p* = 0.48), and 210. 28±89.34 (*P*<0.001) respectively (Figure 3C); and CD86: 156.93±32.67,134.53±26.66 (p = 0.57), 130.86±25.18 (p = 0.55) and 65.18±12.16 (*P*<0.001) respectively (Figure 3D and 4).

### 25(OH)D_3_ Inhibits DC Capacity to Induce T Proliferation

One of the important functions of DCs is to activate and direct T cell responses during an infection. To assess the ability of BMDCs to activate T cells, the BMDCs were cocultured with purified T cells at different ratios for 5 days, and the proliferation of the T cells was monitored. After BCG treatment, the control BMDCs significantly increased the proliferation of T cells, whereas 25(OH)D_3_ and 1,25(OH)_2_D_3_(10 pM)-treated BMDCs had a reduced capacity to stimulate T cell proliferation(*p*<0.05). 1,25(OH)_2_D_3_(100 nM)-treated BMDCs had a significantly reduced capacity to stimulate T cell proliferation(*p*<0.001). This was evident for all the DC to T cell ratios tested (Figure 5).

### 25(OH)D_3_ Inhibits BMDCs Proliferation

The Optical density (OD) value of the −/+25(OH)D_3_, 1,25(OH)_2_D_3_(10 pM) and 1,25(OH)_2_D_3_(100 nM)-treated cultures were 0.61±0.25, 0.54±0.25, 0.49±0.24 and 0.21±0.15 respectively (Figure 6) in the WST-1 proliferation assay, indicating a reduction in the rate of proliferation with all vitamin D metabolites.

### Vitamin D Metabolites Modulate BMDC Cytokine Production

The levels of IFN-γ (Fig. 7 A), IL-2 (Fig. 7 B), IL-6 (Fig. 7D) and IL-10 (Fig. 7 E) were significantly higher and IL-4 (Fig. 7 C) lower in the media from 25(OH)D3 and 1,25(OH)2D3(10 pM)-treated cells when compared to control cells. IL-12 (Fig. 7 F) levels were not different between the 25(OH)D3 and 1,25(OH)2D3(10 pM)-treated cells and control cells. However, the levels of IFN-γ, IL-2, IL-6, IL-10 and IL-12 were significantly lower in the 1,25(OH)2D3(100 nM)-treated cells than control cells, the level of IL-4 was significantly higher in the 1,25(OH)2D3(100 nM)-treated cells than control cells.

### BMDC Expression of the CYP27B1 and VDR

The BMDCs can express the genes of CYP27B1and VDR. The expression of CYP27B1 in 25(OH)D_3_ -treated cells was higher than control-treated cells (Figure 8A) (p<0.05); But not significantly different between 1,25(OH)2D3(100 nM)-treated cells and control-treated cells (*p* = 0.83).

The expression of VDR in 25(OH)D_3_ -treated cells was lower than control-treated cells (Figure 8B) (p<0.05); But not significantly different between 1,25(OH)2D3(100nM)-treated cells and control-treated cells (*p* = 0.62).

## Discussion

Vitamin D deficiency is associated with an increased risk of tuberculosis in various populations [22], [23]. However, the association between *TaqI* and *FokI* VDR gene polymorphisms and susceptibility to tuberculosis is still controversial [24]. Therefore, it is very important to study the relationship between vitamin D levels and TB. TB has evolved to evade innate and adaptive immunity. Numerous study detailed the mechanism of how vitamin D utilizes the innate immune system to clear TB [12], [25]; however, the mechanism governing the effects of vitamin D on the adaptive immune response to mycobacterial infection is less well characterized. Dendritic cells are now recognized as the major effectors that mount the effective T cell response against *M. tb.* Although Penna et al [18] reported that 1,25(OH)_2_D_3_ inhibits the differentiation, maturation and activation of DCs, it is important to understand how physiological concentrations of 25(OH)D affect the DC response as vitamin D deficiency relates specifically to low circulating concentrations of 25(OH)D, not 1,25(OH)_2_D [26]. In this study, we investigated the phenotypic characteristics and functional capabilities of mouse bone marrow-derived dendritic cells (BMDCs) after differentiation in media containing 25(OH)D_3_(100 nM) or 1,25(OH)_2_D_3_ (10 pM or 100 nM) and stimulation with Bacillus Calmette-Guérin (BCG).

First, we found that the percentage of BMDCs that were positive for CD86 was lower in the 25(OH) D_3_-treated cells than in the control cells. The percentages of cells that were positive for other molecules (CD11C, CD80) were not different, and the percentage of cells that were positive for MHCII did not differ between the two treatments. However, the mean fluorescence intensity of MHC II in the 25(OH)D_3_-treated cells was markedly lower than that of the control cells. We also found that 1,25(OH)_2_D_3_ (100 nM) significantly inhibits the expression of CD11C, MHC II, CD80 and CD86, but the expression of CD11C and CD80 is not different between 1,25(OH)_2_D_3_ (10 pM) -treated cells and 25(OH) D_3_-treated cells. CD11C is an important marker used to identify BMDC in mouse, CD86, CD80 and MHC II are costimulatory molecules in differentiated BMDCs. From our results, we can see that 25(OH)D_3_ has no effects on the differentiation of bone marrow cells to DCs, but decrease the expression of MHC II and CD86. Rajashree et al suggested that reduced MHC II expression and variable CD86 expression are common features of the functional impairment occurring in various immunodeficient models [17]. Our results also showed that 25(OH)D_3_-treated cells had a diminished ability to stimulate T cell proliferation.

In addition, treatment with 25(OH)D_3_ affected the proliferation of DCs from bone marrow cells, and 1,25(OH)_2_D_3_ (100 nM) significantly inhibits the proliferation of DCs. Penna et al [18] suggested that 1,25(OH)_2_D_3_ may modulate the immune system by acting at the very first step of the immune response via the inhibition of DC differentiation and maturation into more potent APCs. Gorman [27] also reported that 1,25(OH)_2_D_3_ reduces the number of DC in the skin, resulting in suppressed immunity and, in particular, reduced contact hypersensitivity (CHS) responses. In this study, we found that 25(OH)D_3_ can also decrease the production of BMDCs.

Furthermore, an important function of DCs during the immune response is the production of proinflammatory cytokines. The secretion of IFN-γ, IL-2, IL-4, IL-6, IL-10 and IL-12 was analyzed in DC culture supernatants obtained from 25(OH)D_3_-treated cells and control cells after BCG stimulation. The levels of IFN-γ, IL-2, IL-6 and IL-10 were higher in the media from 25(OH)D_3_ cells when compared with control cells. IFN-γ and IL-2 are essential for protection against mycobacterium [28]. Elevated concentrations of IFN-γ and IL-2 can predominantly activate CD4+ Th1 cells, a T cell subset that plays a critical role in the acquisition of resistance to *M. tb* [29]. IL-6 plays a central role during TB infection [30], and has been used to monitor TB treatment [31].Therefore, 25(OH)D_3_ may strengthen the immune response of Th1 cells to TB infection. Nevertheless, other studies have shown that the Th2 cytokines are also necessary to fight TB infection. As an anti-inflammatory cytokine, IL-10 has been hypothesized to balance the macrophage environment that is dominant in the lungs and lymph nodes [32]. Previous laboratory findings reported that enhanced IL-10 secretion by cells of chronic TB patients may impede a rapid and effective Th1 type anti-mycobacterial response [33], and IL-10 has been identified as a cytokine that is most important for preventing an excessive Th l response both systemically and in granulomas [34]. Our results indicate that 25(OH)D_3_ may regulate the balance between Th1 and Th2 cells during TB infection.

In contrast, IL-4 levels were lower in 25(OH)D3–treated cultures than in control cultures. IL-4 is the most important factor for development of the Th2 phenotype [35]. Experimental murine infection has proven that increased production of IL-4 during the chronic phases of infection is characterized by progressive fibrosis and necrosis. Later publications have suggested that the increased production of IL-4 by CD4+ cells, as well as by CD8+ T cells in TB patients and especially in patients with cavitary TB, proved to play a role in tissue necrosis [36]. 25(OH)D_3_ down-regulates the expression of IL-4, which may decrease tissue fibrosis and necrosis.

IL-12 is the most important factor in Thl phenotype development [35]. Because mycobacteria are extremely strong IL-12 inducers, *mycobacterial* infection can skew the response to a secondary antigen toward a Th1 phenotype [37]. IL-12 is a crucial cytokine for the control of *M. tuberculosis* infection. The exogenous administration of IL-12 to BALB/c mice can improve survival [38], and IL-12−/− mice are very susceptible to *M. tuberculosis* infection [39]. In our study, we found that 25(OH)D_3_ has no effect on the level of IL-12 secreted by BMDCs, but 1,25(OH)_2_D_3_ inhibits the secretion of IL-12. Hewison et al [20] also reported that 1,25(OH)_2_D_3_ inhibits the secretion of IL-12 in human monocyte-derived dendritic cells.

25(OH)D_3_ up-regulated the levels of IFN-γ, IL-2, IL-6 and IL-10; however, it down-regulated the secretion of IL-4, these data are contrary to 1,25(OH)_2_D_3_, which inhibits the secretion of IFN-γ, IL-2, IL-6, IL-10 and IL-12, while inducing IL-4. One reason may be that vitamin D may regulate the immune response of BMDC by controlling the expression of CYP27B1 and VDR. Physiological levels of 25(OH)D and 1,25(OH)2D are pro-inflammatory, but supra-physiological levels are anti-inflammatory. One reason why 100 nM 1,25(OH)2D– treated cells produce less pro-inflammatory cytokines is also likely due to the fact that we have shown these cells are much less differentiated compared to the other cells (morphologically and on flow cytometry). Therefore, interpretation of results with supra-physiological levels must be taken with caution as physiological levels can induce an opposite effect.

Th1 and Th2 cell types regulate each other via their cytokine synthesis, which is important in the final balance of host resistance against pathogens [29]. The interplay between Th1 and Th2 lineages to maintain the Th1/Th2 balance is a crucial immune mechanism of the host to clear bacteria. In murine models of TB, Th1 and Th2 cytokines are reported to play opposing roles in the defense against TB [40]. During the past decade, increasing evidence has indicated the need for Th1-type responses for the immediate containment of the TB infection; in contrast, Th2-type responses are necessary for late-phase granuloma formation [34]. Previous investigations of immune responses to TB infections have shown a significant correlation between abnormal characteristics of Th1 and Th2 cytokine expression and the severity of the disease [41]. Thus, it is important to produce enough of a proinflammatory type 1 response to keep the intracellular infection under control while concurrently producing just enough of a type 2 response to prevent the type 1 response from causing damage to the host. This requires an extremely careful “dosing” of either type of response and tight control over where and when type 1 and type 2 responses occur [35].

In conclusion, during *mycobacterial* infection, 25(OH)D_3_ affects the proliferation of DCs, the expression levels of MHC-II and CD86 on the surface of the DCs and the ability of the DCs to stimulate proliferation of allogeneic mixed lymphocytes. Furthermore, it decreases the level of IL-4 secreted by the BMDCs, but increases the levels of IL-2, IL-6, IL-10 and IFN-γ. These findings suggest that 25(OH)D_3_ modulates the immune response during *mycobacterial* infection by affecting the maturation and function of DCs. However, it remains to be determined whether the same effects of physiological 25(OH)D will occur in human DC during TB infection in vivo and in vitro. Understanding the role of 25(OH)D during TB infection may aid in the development of novel strategies for the treatment of tuberculosis.
